# The Impact of Injury on Career Progression in Elite Youth Football—Findings at 10 Years

**DOI:** 10.3390/jcm13071915

**Published:** 2024-03-26

**Authors:** Yannic Bangert, Ayham Jaber, Raphael Trefzer, Severin Zietzschmann, Kevin-Arno Koch, Ralph Kern, Jan Spielmann, Tobias Renkawitz, Johannes Weishorn

**Affiliations:** 1 Department of Orthopaedics, Heidelberg University Hospital, Schlierbacher Landstrasse 200a, 69118 Heidelberg, Germany; yannic.bangert@med.uni-heidelberg.de (Y.B.); ayham.jaber@med.uni-heidelberg.de (A.J.); severin.zietzschmann@med.uni-heidelberg.de (S.Z.); kevin-arno.koch@med.uni-heidelberg.de (K.-A.K.); tobias.renkawitz@med.uni-heidelberg.de (T.R.); 2 TSG 1899 Hoffenheim Fußball-Spielbetriebs GmbH, Horrenberger Straße 58, 74939 Zuzenhausen, Germany; ralph.kern@tsg-hoffenheim.de; 3 Ethianum, Fehrentzstrasse 2, 69115 Heidelberg, Germany; 4 TSG ResearchLab gGmbH, Horrenberger Straße 58, 74939 Zuzenhausen, Germany; jan.spielmann@tsg-researchlab.de

**Keywords:** adolescent, career progression, football, soccer, elite youth academy, sports medicine

## Abstract

**Background**: There is a lack of evidence regarding the impact of time loss, match exposure, and age at injury on career progression in elite football. Therefore, the aim of this study was to identify injury characteristics and their influence on career progression in a German youth academy. **Methods**: During the 2012/2013 season, a prospective cohort study reported 107 time-loss injuries among 130 young athletes from an elite German soccer academy. Individual career progression was analyzed using 10-year data. **Results**: Injuries and time loss were not associated with career progression (*p* > 0.05) in the overall cohort. In the U17 and U19 groups, 24% were able to reach the professional level, with injuries significantly decreasing this probability (*p* = 0.002). Injuries lasting more than 28 days had a negative impact on career progression compared to minor injuries (30% vs. 10%; *p* = 0.02). **Conclusions**: Not only the characteristics of injuries, but also their impact on career development, vary with age. In the U17 and U19 age groups, serious injuries resulting in more than 28 days of absence have a negative impact on career progression. It is important to be aware of these effects in order to focus on the prevention of long-term injuries to ensure the optimal development of young athletes.

## 1. Introduction

Football (soccer) is the world’s most popular sport, played by an estimated 270 million people worldwide [1]. Although only a small proportion of young players actually pursue a professional career, there is a strong desire to do so. In addition to talent and hard work, it is important to prevent injuries in order to minimise time loss and maximise playing time. Injuries in elite youth football result in reduced involvement in training and play. Therefore, injury avoidance is an important factor in the optimal career progression of young athletes [2,3]. Although there have been a number of recent studies on the characteristics and patterns of injury in elite international youth academies, there has been very little research on the impact of injury on long-term career progression [4,5,6].

Established measures of injury, such as injury incidence or injury burden, are often considered holistically, ignoring the resulting individual consequences for the athlete [7]. It is known that injuries can lead to young athletes being unable to continue their careers as desired due to the adverse consequences [8]. After anterior cruciate ligament (ACL) repair, between 77% and 95% are able to return to their sport, but only 65% are able to return to their previous level of play [9]. Young athletes pass through an intense and dynamic maturation process in adolescence, with fragile structures and periods of growth [10,11]. It is one of the explanations suggested for the elevated rates of age-related injuries and the increased injury burden during the period when body size and weight change the most [6,12,13,14,15]. The development of young footballers is essentially divided into the sampling phase (between the ages of 6 and 12), the specialisation phase (between the ages of 12 and 16), and the investment phase (between the ages of 17 and 19), in which the leap to the professional level is to be made [16]. The extent to which the time lost due to a severe injury, or the cumulative time lost due to several minor or moderate injuries, affects the individual career progression of elite youth football players has not yet been investigated. Furthermore, the extent to which time loss, match exposure, and age at injury affect individual development has not yet been investigated.

The aim of the present study was, therefore, to describe the injury burden of the most common injury types in an elite youth football academy and to investigate the individual influence of time loss, match exposure, and age at injury on career progression.

## 2. Materials and Methods

This study analyses injury data from a cohort of 138 male players in an elite U12–U19 academy in Germany during the 2012/13 season and relates these to professional career progression in the decade following the index season. The study was conducted in accordance with the Declaration of Helsinki and approved by the Institutional Review Board of the University of Heidelberg (S—254/2023). The injury data was collected with the consent of the players and their guardians. 

**Figure 1:**
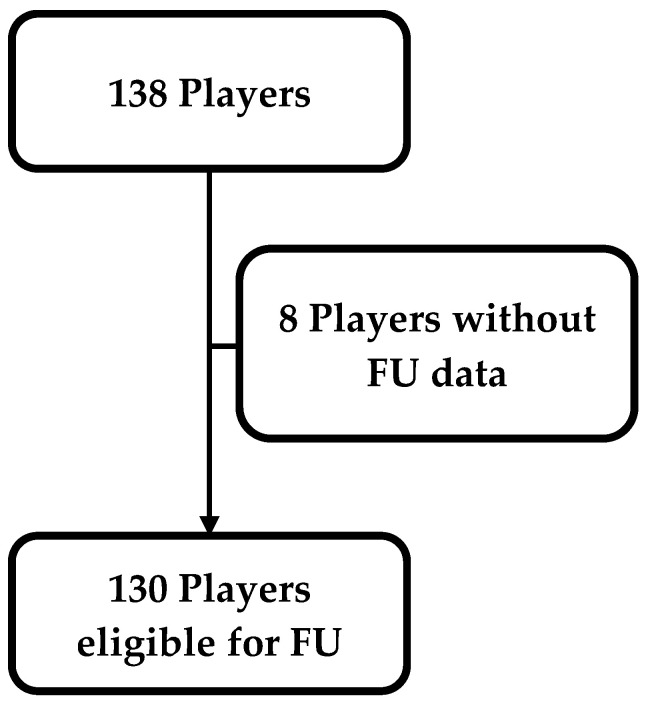
Flowchart visualizing player selection at the current 10-year FU.

Criteria for inclusion were club membership throughout the 2012/13 season, complete injury registration, and full recording of training and match exposure throughout the season. A club-specific database was used to monitor the individual career development of each player. Players without individual career data at 10 years were excluded from the statistical analysis (Figure 1). The index season started with the pre-season in July 2012 and ended in May 2013. To present the observational results of this study, the Strengthening the Reporting of Observational Studies in Epidemiology (STROBE) statement was applied [17].

The Academy implements a structured training regime that focuses on both physical fitness and football-specific skills. Training frequency varies across age groups, with younger players (U12 and U13) typically attending an average of three sessions per week, while older players (U16, U17, and U18) typically attend an average of five sessions per week. Match attendance also varies, ranging from 44 matches for U16 players to 74 matches for U13 players. Match duration varies from 60 min for U12 and U13 matches to 90 min for U19 matches. U19 players accumulate the highest match exposure, with a total of 891 h. Importantly, the study did not include any additional training or matches associated with junior national teams, and the participants were not associated with the interpretation or design of the study.

### 2.1. Injury Surveillance

All players were instructed to promptly notify the club’s medical staff in the event of any injury. Upon notification, a doctor or physiotherapist conducted a thorough medical examination and, if necessary, prescribed relevant diagnostic tests to ascertain the nature of the injury. Following the methodology outlined by Fuller et al., details, including the affected type of injury, body region, and its classification as overuse or acute, were meticulously documented in a spreadsheet database [18]. 

Although the exact mechanism of the trauma couldn’t always be determined, the diagnosis was made through a collaborative effort between physiotherapists and physicians in an interprofessional exchange. Throughout this process, players and coaches were actively engaged and fully informed about the injury situation. Dates of injury occurrence and return, defined as the point of complete recovery for training or competition, were meticulously recorded. The rehabilitation progress was diligently monitored until the completion of the rehabilitation protocol. In instances of persistent injuries, the treating clinician estimated the expected return date at the conclusion of the observation period [5]. The severity of injuries was categorised into three groups: mild (resulting in 4–7 days of activity loss), moderate (resulting in 7–28 days of activity loss), and severe (resulting in more than 28 days of activity loss). Minor injuries with less than 3 days of activity loss were not included in the records. To ensure data accuracy, injury records were reviewed weekly for completeness. Instances of non-sport-related time loss, such as general illness, were excluded from the study analysis.

### 2.2. Exposure and Career Surveillance

A standardised injury record form [19] was used to record training exposure and lost time injuries for each player (Table 1). A list of injury definitions was included with the injury record form to improve consistency among data collectors. All musculoskeletal injuries were methodically documented according to the definitions and guidelines outlined by Fuller et al. [18]. Exposure data from training sessions and matches for the U17 and U19 teams were electronically recorded by the coaches, while, for the U12 to U16 age groups, the team physician and medical staff were responsible for recording exposure data. Match exposure was measured at the individual level, while training exposure was measured at the team level.

To evaluate individual player profiles, data from the club’s internal database were analysed. A professional football player was defined as a full-time athlete with a contract, salary, and full insurance in a national or international professional football league.

### 2.3. Data Processing

Descriptive statistics were used to describe the data. Parametric data were expressed as mean and standard deviation, and skewed data as median and interquartile range. Frequencies were expressed as absolute and relative numbers, and comparisons between groups were made using the chi-squared test, with significance set at a *p*-value of less than 0.05. Visual presentation of results was provided through graphical illustrations, where appropriate. Statistical analysis and graphs were performed using Microsoft Excel version 2308 (Microsoft, Redmond, WA, USA) and SPSS 26.0 (IBM, Armonk, NY, USA).

Injury incidence was calculated by determining the number of injuries per 1000 h of exposure, reported both overall and by injury type [6]. Considering the non-parametric distribution, the median was utilised to calculate the specific injury burden, defined as the product of the number of injuries and the injury severity [6]. The injury burden was quantified as the number of days lost per 1000 h of exposure, while the variability was reported as the 95% confidence interval based on a Poisson distribution [6]. Detailed operational definitions used throughout the study are provided in Table 1.

## 3. Results

A total of 130 players were assessed. The mean age of the participants was 15.0 years, with a standard deviation of 2.1 years. During the observation period, the cumulative exposure to football activities was 40,120 h, and the average season length was 40 weeks, ranging from 35 to 45 weeks.

### 3.1. Main Injury Outcomes

During the 2012/13 season, the cohort suffered a total of 107 injuries. On average, players missed approximately 23 days of training or competition as a result of these injuries. Each player suffered an average of 0.82 injuries per season. The average injury incidence rate was calculated to be 2.7 injuries per 1000 h of exposure. Furthermore, the injury incidence rate among players with at least one injury was determined to be 50% (65/130 players). Table 2 provides a summary of demographics, exposure, and injury characteristics categorised by age.

The total absence time in the study cohort was 2474 days, resulting in a total injury burden of 62.4 days (41.6 to 83.2, 95% CI) per 1000 h of exposure. The mean severity of injury was 23.1 days (15.4 to 30.8, 95% CI). 

Joint sprains were the most common injury type, with 31 injuries. This was followed by muscle injuries (n = 30), bone fractures or stress reactions (n = 12), physeal injuries (n = 12), and contusions (n = 12). Bone and physeal injuries were associated with the highest mean injury severity. Joint sprains and bone injuries were associated with the highest injury burden in the youth academy studied (Table 3).

**Figure 2:**
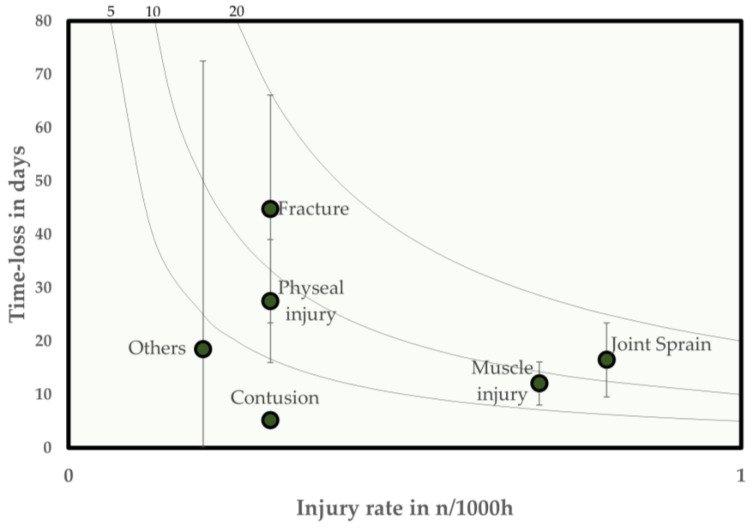
Risk matrix showing the total burden of injury for different types of injury in elite youth football players—the burden of injury is shown as the product of the incidence and the severity of injury. Error bars show 95% CI. Isobars indicate injury burden in terms of 5, 10 and 20 days lost per 1000 h of exposure.

The injury burden as a product of injury incidence and severity is also shown as a risk matrix in Figure 2. Isobars for injury burdens of 5, 10, and 20 days lost per 1000 h of exposure are used for reference. 

### 3.2. Impact of Injury on Career Progression

In the 2012/13 season, a total of 107 injuries were recorded among 130 youth players in the study cohort. Of these, 25 players (19%) made the transition to professional football after 10 years, while 49 others are currently playing in lower leagues.

Players who later signed a professional contract suffered a comparable number of injuries in the 2012/13 season (0.9 ± 1.1 vs. 0.8 ± 1.1 injuries; *p* = 0.7) and lost a comparable amount of time (21.7 ± 47.8 vs. 18.4 ± 42.0 days; *p* = 0.6) as players who did not make the transition to professional football. In addition, the number of injuries a player sustained had no effect on individual career progression (*p* = 0.9).

Looking at the U17 and U19 groups in more detail, it can be seen that although the professional players have a comparable injury frequency (1.1 ± 1.4 vs. 1.3 ± 1.3 injuries; *p* = 0.7), the average time lost per season is substantially lower (10.4 ± 14.0 vs. 33.3 ± 64.1 days; *p* = 0.067) than in the comparison group. 

Injured U17 and U19 players received less playing time in 2012/13, were more likely to be dropped from the squad, and were significantly less likely to go on to pursue a professional career (Table 4).

In this age group, the chances of having a professional career decrease as the severity of the injury increases (Table 5).

**Figure 3:**
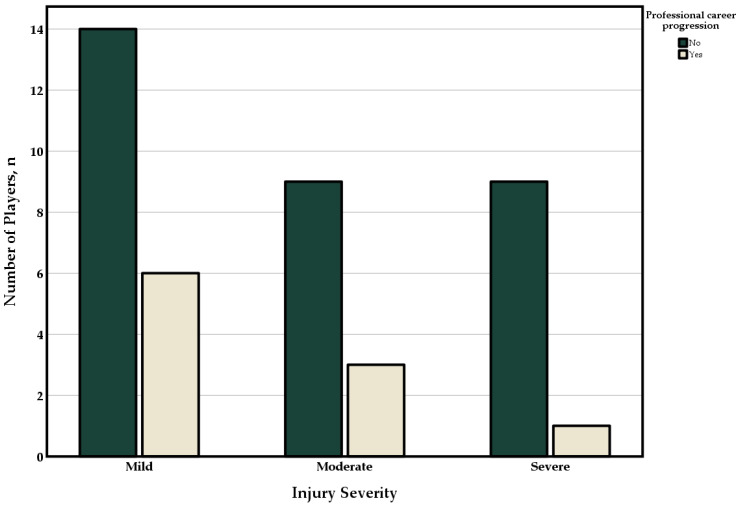
Career progression by injury severity.

In the group of players with no or only a minor injury in the investment stage, 30% of the players reached the next step and were able to start a professional career. The proportion was significantly lower for players who suffered a serious injury with >28 days of absence (*p* = 0.024, χ^2^ = 1.5; Figure 3).

## 4. Discussion

In developing young athletes, lost exposure due to injury leads to a potential loss of specific development. This is difficult to compensate for and often results in reduced individual support from their club [22]. The characteristics of injuries vary between populations and age groups, and little is known about their impact on career progression. It is therefore important to provide national evidence.

The characteristics of age-related injuries in the German elite youth academies have been published recently [23]. The objectives of this study were, therefore, to describe the injury burden of the most common injury types in an elite youth football academy and to investigate the individual influence of time loss, match exposure, and age at injury on career progression.

### 4.1. Main Injury Outcomes

The literature on elite international youth academies provides a wide range of data on injury incidence. These range from 1.3 to 21.1 injuries per 1000 h of exposure [2]. In a unique cohort of young academy footballers in England, Price et al. found an average time loss of 22 days per injury. Similarly, Materne et al. found a mean time loss of 19 days in youth footballers from Qatar [12,24]. Differences observed in the literature, particularly in terms of injury incidence, can be explained by structural differences between different international youth academies [15,25,26]. The injury incidence for the present cohort of elite youth players from a German academy was 2.7 injuries per 1000 h of exposure, resulting in an average time loss of 23.1 days. This is lower but comparable to the incidence rates of 4.3–5.8 per 1000 h for adults in the UEFA Champions League and in Swedish professional football [27]. The highest overall injury burden in the present study was observed for joint sprains (13.2, 7.6 to 18.7) and fractures (13.4, 7 to 19.8). It is particularly interesting to note that, although fractures accounted for only 11% of the injuries in the collective analysed, they had a comparable injury burden to joint sprains, which accounted for a third of all injuries. Nevertheless, the proportion of fractures in the collective studied is quite high compared to the 2–9% reported in the literature [2].

Injuries to the joint capsule and ligaments were common and of moderate severity. Muscle injuries represented 29% of all injuries, which is comparable to the 31% reported in professional football [28]. Muscular injuries were associated with an average absence of 8.6 days per 1000 h of exposure. The U19, U16, and U15 age groups showed a particularly high incidence and severity of injuries, particularly those affecting the thigh muscles, which pose a significant challenge [29,30]. The quest to reduce hamstring injuries has led to the creation of Nordic Hamstring Training, which has shown significant effectiveness in decreasing hamstring injuries [31]. The effectiveness of alternative programs like plyometric training remains a topic of debate in the literature [30]. Despite the existence of validated prevention strategies, possibly due to a lack of awareness and reluctance to participate in specific programmes, hamstring injuries continue to increase in both professional and amateur football [28,32]. Increased physicality, as reflected in high-intensity sprints, tackles, and volume of sprints, is also considered a contributing factor [33]. These worrying trends require further research and treatment.

Apophyseal injuries were a significant risk factor for prolonged absence, accounting for 11% of all injuries and 28% of all serious injuries. Comparable injury rates of 0.4 to 4.7 injuries per squad season have been reported [6,12,14]. In recent years, the focus of injury prevention in young athletes has shifted to physeal injuries [5,23]. These injuries occur predominantly in the age groups between U13 and U15. Therefore, the extent to which they have an impact on career progression should be discussed in light of the results of the present study.

### 4.2. Impact of Injury on Career Progression

Playing football in an elite youth academy, with all its professional processes, high-level coaching, and individual attention, does not necessarily lead to a professional career for the young athlete. There is still a long way to go before a player can become established in an elite national or international league. In order to reach the elite level as an adult, a maximum of playing time, especially in the last years of youth, the so-called investment phase, is discussed [16]. Injuries, on the other hand, require time lost and may, therefore, play a crucial role in the vulnerable developmental phase of elite young footballers [2]. However, knowledge of the impact of injury and time loss on individual players and their career progression is generally poorly understood. To date, moderate evidence exists almost exclusively for ACL injuries and is mostly based on data from players who have already made the leap to the professional level. It has been shown that 77–95% of athletes with a previous ACL repair return to sport. However, only 50–65% are able to play their sport at the same level 3 years after surgery [9,34,35,36].

In the short term [34], a significant decline in player performance was observed. Good performance during the investment phase is particularly important for a young player. It is important to know whether an ACL injury can lead to a return to peak performance. It is also important for physicians to know this in order to advise on therapy. In this study, we were able to show that injuries generally do not have a negative effect on the overall career progression of athletes in an elite youth academy. However, we were able to show that this conclusion does not necessarily apply to all age groups. In the investment phase of U17 and U19 players, there was a negative effect on career progression in the following decade. In this age group, just under 24% made the step to the professional level. Similar data can be found in the literature, with just under 30% of this age group making the transition to the professional leagues [37]. In the present study, we were able to show that players who suffered a serious injury with more than 28 days lost during this vulnerable career phase were significantly less likely to achieve a professional career (10%) than players who suffered a minor injury (30%) or no injury (36%). Therefore, in order to achieve a smooth development of athletes, the focus of injury prevention in this selected age group should be placed on physeal and bone injuries, as they are associated with the longest median time loss.

There are other factors, besides injury, that determine the transition to the elite professional level. Factors such as performance level, consistency of performance, rate of performance improvement, long-term participation in a youth national team, and resilience to physical and psychological stress have been described in the literature [38,39]. Career progression also appears to be influenced by the relative age effect [40,41]. The relative age effect is the phenomenon that athletes born in the first half of the selection year are overrepresented in professional sports. In the German youth national teams as well as in the German Bundesliga, such players benefit from this physical advantage mainly at a young age [40]. Similar effects have also been observed in the youth national teams of other nations [41]. However, this effect decreases with age. In adult professional soccer, it plays only a minor role [40,41]. It may also be interesting to examine players who did not make the transition to professional football in order to better understand the factors that influence individual career progression and the transition to professional football.

### 4.3. Strength and Limitations

Several limitations need to be discussed. The exclusive focus on a single elite football academy in Germany limits the generalisability of the findings. To improve external validity, future studies could adopt registry structures similar to those used in other sports orthopaedic subspecialties [42]. However, differences in training methods, coaching techniques, and match formats between clubs may limit the comparability of injury rates. In addition, confidentiality concerns among professional football clubs may hinder efforts to share data [43]. As a result, longitudinal studies over multiple seasons within a single academy may provide a more insightful approach. 

Throughout this study, all academy and club activities were meticulously documented over the course of the season. However, it’s important to note that there was no record of unscheduled activities, and any exposure to the national team was not recorded. The occurrence of injuries, particularly those of a gradual nature, may have been influenced by these unobserved factors. Furthermore, when interpreting and applying the conclusions drawn from this study, it’s important to recognise the influence of contextual factors such as training philosophies, lifestyle habits, and environmental conditions.

In addition, it is important to understand that there are other factors besides injury that may influence an athlete’s individual career trajectory. Factors such as training, coaching, socioeconomic background, education, and personal circumstances may play an important role in an athlete’s career trajectory [40]. However, these factors are difficult to operationalise and are therefore not part of the analysis and interpretation of this study. However, it is important to be aware of the multifactorial nature of elite youth athlete development when interpreting the results. It is also noteworthy that the study design limits the ability to account for potential pre-existing or subsequent injuries outside the study period that may have influenced the athlete’s career trajectory.

Furthermore, a critical discussion of the primary objective of the study, the attainment of elite level in adult football, is necessary. Similar studies have used additional performance measures to determine return to performance [35]. These and other individual athlete data were not available due to club transfers, failure to transition to the professional level, or club restrictions on access to the study authors due to sensitive content, thus limiting the power of the study.

However, this cohort study of elite youth footballers is the first of its kind to correlate the injury characteristics of athletes from an elite youth academy in Germany with their individual career progression. As recently emphasised, this is a substantial addition to the aspects of injury research in elite athletes [20].

## 5. Conclusions

While injury characteristics such as injury incidence, injury burden, and injury patterns have been increasingly studied in the literature and the understanding of vulnerable phases in the development of youth players has recently been characterised, the impact of injuries on individual career progression has been scarcely studied. This prospective study of an elite youth academy in Germany is the first to demonstrate a direct impact of injuries on the career development of individual players over the following decade. In the U17 and U19 age groups, 24% were able to reach the professional level, with injuries significantly reducing this probability. In particular, injuries lasting more than 28 days had a negative impact on career progression in this age group. Given the high financial investment made by clubs and national federations in the development of players, a high transition rate to the professional level is desirable. Among other factors, the loss of training and match exposure due to injury should not be underestimated in order to improve the transition rate. Longitudinal studies or analyses of unified registries that consider injuries over multiple seasons and capture additional factors, such as resilience to physical and psychological stress, are needed to further investigate the findings of this study.

## Figures and Tables

**Table 1:** Definitions used in the study.

Measure	Definition
Player season	A player who played in a given season.
Time-loss injury	A physical condition or symptom observed in a player that necessitates the intervention of medical staff to restrict the player’s involvement in either full or partial participation during an upcoming football team training session or match [18].
Injury incidence	Frequency of injuries with time loss per 1000 playing hours [18].
Injury burden	The impact of injury, calculated as the total number of days lost per 1000 playing hours, as a product of frequency and severity [20].
Muscle injury	Injuries with functional damage, structural damage or full-muscle rupture corresponding to class II to IV, as defined by Müller-Wohlfahrt et al. [21].
Bone injury/Fracture	Structural damage to the bone, such as oedema, stress fractures, periostitis, or fractures caused by trauma.
Joint sprain	Strained or torn ligaments responsible for joint stability or the joint capsules themselves.
Physeal injury	Physeal injuries (e.g., apophysitis) or physeal fractures

**Table 2:** Demography, Exposure, and Injury characteristics.

	U12	U13	U14	U15	U16	U17	U19
Players (n)	14	17	18	18	21	21	21
Age (y, SD)	11.7 (0.4)	12.8 (0.5)	13.4 (0.4)	14.5 (0.5)	15.6 (0.5)	16.6 (0.5)	18.0 (0.8)
Stature (cm, SD)	149.0 (7.5)	157.5 (7.1)	162.1 (7.3)	169.9 (7.0)	173.6 (6.3)	176.7 (5.2)	181.7 (7.8)
Body mass (kg, SD)	42.5 (5.4)	44.9 (6.0)	49.0 (5.6)	55.9 (6.0)	62.4 (6.1)	69.8 (6.7)	75.1 (7.5)
Time-loss injuries (n)	4	4	13	12	22	20	32
Total time lost (days)	38	86	294	257	629	148	1022
Overall injury incidence	1.4	1.2	2.6	2.3	2.8	2.6	4.0
Mean days lost per injury (95% CI)	9.5 (3.6–15.4)	21.5 (0–51.7)	22.6 (7.2–37.9)	21.4 (6.4–36.5)	28.6 (10–46.8)	7.4 (5.2–9.6)	31.9 (10.3–53.6)
Injury burden (95% CI)	13.3 (5.0–21.6)	25.8 (0–52.0)	58.8 (18.7–94.9)	49.2 (14.7–84)	80.1 (28–131)	19.2 (13.5–25)	127.6 (41.2–214.4)

**Table 3:** Injury incidence, severity, and burden by injury type.

	Injuries Incidence Rate		Median Time Loss	Burden
Injury Type	n	Injuries per 1000 h	Days (IQ 25th–75th)	Time-loss days per 1000 h (95% CI)
Joint sprain	31	0.8	16.5 (9.5–23.4)	13.2 (7.6 to 18.7)
Muscle injury	30	0.7	12.1 (8–16.1)	8.5 (5.6 to 11.3)
Bone injury/fracture	12	0.3	44.8 (23.4–66.1)	13.4 (7 to 19.8)
Physeal injury	12	0.3	27.5 (16–39)	8.3 (4.8 to 11.7)
Contusion	12	0.3	5.2 (4.2–6.1)	1.6 (1.3 to 1.8)
Others	10	0.2	18.5 (0–72.5)	3.7 (0 to 14.5)

**Table 4:** Playtime and match participation in U17 and U19 teams stratified by injury history.

	Injured		*p*-Value
	Yes	No	
Total, n	28	14	
BMI, kg/m^2^	23.2	22.5	0.9
Playtime in min., mean (SD)	877 (114)	1085 (186)	0.3
Matches in the squad without an appearance, mean (SD)	3.3 (0.7)	2.0 (0.7)	0.3
Matches not listed in squad, mean (SD)	8.5 (1.3)	5.7 (1.5)	0.2
Time loss, mean (SD)	41.8 (12.4)	0 (0)	**0.002** *
Chances of a professional career	5/28 = 17.9%	5/14 = 35.7%	**0.02** *

* indicates significance.

**Table 5:** Probability of professional career in U17 and U19 teams based on severity of injury.

Injury Severity	Probability of a Professional Career
Overall	10/42 = 23.8%
Mild (<7 days)	6/20 = 30%
Moderate (7–28 days)	3/12 = 25%
Severe (>28 days)	1/10 = 10%

## Data Availability

The data presented in this study are available on request from the corresponding author. The disclosure of sensitive data of professional footballers may be prohibited by the participating club.
